# A Preliminary Study on the Presence of *Salmonella* in Lymph Nodes of Sows at Processing Plants in the United States

**DOI:** 10.3390/microorganisms8101602

**Published:** 2020-10-18

**Authors:** Roger B. Harvey, Keri N. Norman, Robin C. Anderson, David J. Nisbet

**Affiliations:** 1 Food and Feed Safety Research Unit, Agricultural Research Service, U.S. Department of Agriculture, College Station, TX 77845-4988, USA; robin.anderson@usda.gov (R.C.A.); david.nisbet@usda.gov (D.J.N.); 2 Department of Veterinary Integrative Biosciences, College of Veterinary Medicine and Biosciences, Texas A&M University, College Station, TX 77843, USA; KNorman@cvm.tamu.edu

**Keywords:** lymph nodes, *Salmonella*, antimicrobial resistance, prevalence, sows, pork

## Abstract

*Salmonella*-contaminated lymph nodes (LN), when included into edible meat products, are a potential source of *Salmonella* foodborne disease. In this survey, ventral superficial cervical and mandibular LN were tested for the presence of *Salmonella* from two sow processing plants in the midwestern United States. Results indicate that both LN can be contaminated with *Salmonella*; mandibular LN have higher prevalence (*p* < 0.05) of *Salmonella* than cervical LN (16% vs. 0.91%), and the majority (>90%) of *Salmonella* isolates are pan-susceptible or resistant to one antimicrobial, while 9.78% of isolates were multi-drug-resistant (MDR-resistant to three or more classes of antimicrobials). Intervention methods to prevent foodborne disease could include elimination of these LN from pork products or inclusion of LN only into products that are destined for cooking. Integrated multi-faceted intervention methods need to be developed to reduce *Salmonella* in the food chain.

## 1. Introduction

The *Salmonella enterica* (hereafter called *Salmonella*) is one of the top five foodborne pathogens globally and a leading cause of foodborne illness in humans [1]. *Salmonella* is reported to cause over 1.3 million infections annually in the United States (U.S.) with approximately 1 million traced back to consumption of contaminated food [2]. These health concerns have prompted the beef, pork, and poultry industries to continuously search for mechanisms to reduce the risk of *Salmonella* contamination of their products. It has been suggested that cattle lymph nodes (LN), when included into beef ground products, could contribute to *Salmonella* contamination [3]. Indeed, peripheral LN of cattle are in anatomic locations that their inclusion into beef products could be somewhat common. The prevalence of *Salmonella*-positive subiliac LN in U.S. cattle are reported to be 7.7% to 62% [4,5,6,7], whereas prevalence of *Salmonella* in inguinal LN of cattle in Mexico are 54–75% [6,8]. Of increasing concern is that 8% to 20% of *Salmonella* isolates in those studies were resistant to antimicrobials [5,7]. 

It is not unreasonable to believe that swine LN, when included in edible pork products, could serve as a source of *Salmonella* contamination for pork. *Salmonella*-positive inguinal LN of sows at slaughter are reported to range from 4.8% to 37% [9,10,11] in the U.S., whereas popliteal LN in market hogs is reported to be 12.6% [12]. A study in Mexican swine abattoirs reported that subiliac LN had a *Salmonella* prevalence of 10% to 20% [13]. There has been limited research on prevalence of *Salmonella* in swine LN. *Salmonella*-positive LN represent a risk of contamination to pork products when incorporated into pork products, such as sausage, chorizo, and others [13,14]. The objective of this preliminary survey was to determine the presence of *Salmonella* in ventral superficial cervical LN and mandibular LN of sows at processing.

## 2. Materials and Methods 

### 2.1. Sample Collection

Two sow processing plants (Plant A and Plant B) in two separate states in the midwestern U.S. were chosen for sampling. Each plant was sampled on two consecutive days each month for twelve months (November 2016–October 2017). Samples consisted of 25 ventral superficial cervical LN on each of days 1 and 2, the first Monday and Tuesday of each month for both Plants A and B (50/month X 12 months = 472 samples for Plant A and 548 samples for Plant B). In addition, from February–October 2017 (9 months), the same day each month that cervical LN was sampled, we collected 150 mandibular LN from Plant A and 217 LN from Plant B (25/day/month/plant X 9 months = 367). Due to weather and other sampling difficulties, we were unable to collect the full number of samples (both cervical and mandibular) as projected from the original study design.

### 2.2. LN Processing, Salmonella Cultivation

LN samples were processed as previously described for meat samples [15]. Tissues were individually weighed and recorded, surface sterilized by submersion in boiling water for 3–5 s, placed into sterile filtered-stomacher bags (Nasco, Fort Atkinson, WI, USA), macerated by pounding with a rubber mallet until flat, enriched in 80 mL of tryptic soy broth (Becton Dickinson, Sparks, MD, USA), and homogenized for 30 s with a laboratory blender (BagMixer 400VW, Interscience Laboratories Inc., Weymouth, MA, USA) at a medium speed (seven paddle strokes per second). The homogenized samples were incubated at 25 °C for 2 h, 42 °C for 12 h, and then held at 4 °C, for no more that 4–6 h, until further processing. For prevalence analysis, 1 mL from each enrichment culture was subjected to anti-*Salmonella* immunomagnetic separation (IMS). Each 1 mL aliquot received 20 µL of anti-*Salmonella* beads (Invitrogen, Carlsbad, CA, USA) and was incubated with shaking at room temperature for 15 min. The beads were extracted from the enriched samples and washed twice in PBS-Tween 20 (Sigma, St. Louis, MO, USA). The beads were transferred to 3 mL of Rappaport Vassiliadis soya (RVS; Remel Products, Lenexa, KS, USA) broth and incubated at 42 °C overnight. *Salmonella* present in these samples were detected by swabbing the RVS enrichment culture onto brilliant green agar (Becton Dickinson, Sparks, MD, USA) containing sulfadiazine (80 mg/L; Sigma, St. Louis, MO, USA). All plates were incubated at 37 °C for 18 to 20 h. After incubation, up to three presumptive *Salmonella* colonies were selected and confirmed biochemically with lysine iron and triple sugar iron agars according to the manufacturer’s recommendations (BD Difco, Dickinson and Company, Sparks, MD, USA). 

### 2.3. Antimicrobial Susceptibility Testing

Susceptibility to 14 antimicrobial agents (cefoxitin (FOX), azithromycin (AZI), chloramphenicol (CHL), tetracycline (TET), ceftriaxone (AXO), amoxicillin/clavulanic acid (AUG2), ciprofloxacin (CIP), gentamicin (GEN), nalidixic acid (NAL), ceftiofur (XNL), sulfisoxazole (FIS), trimethoprim/sulfamethoxazole (STX), ampicillin (AMP), and streptomycin (STR)) was determined by use of an automated micro-broth dilution method (Sensititre Gram Negative NARMS Plates (CMV3AGNF), TREK Diagnostics Inc., Cleveland, OH) according to the manufacturer’s recommendations. Isolates were classified as susceptible, intermediate, or resistant using breakpoints established by the Clinical and Laboratory Standards Institute [16]. Isolates that were resistant to three or more classes of antimicrobials, as defined by the National Antimicrobial Resistance Monitoring System (NARMS), were considered multi-drug resistant (MDR). 

### 2.4. Statistical Analysis 

Descriptive statistics comparing *Salmonella* prevalence across LN, plants, month, and day were tabulated in Stata (version 16, Stata Corp, College Station, TX, USA). Logistic regression (Stata version 16) was used to explore associations between *Salmonella* prevalence within and across LN for plant, month, and day (*p* < 0.05). 

## 3. Results and Discussion

**Figure 1:**
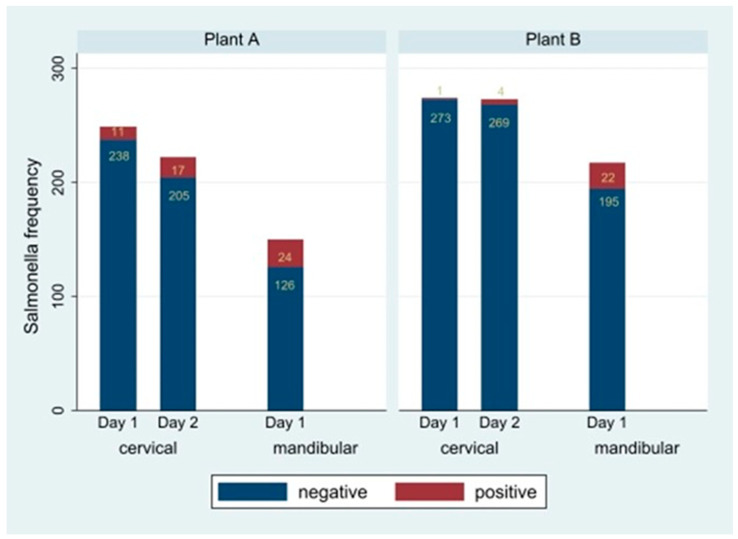
Prevalence of *Salmonella* in lymph nodes of sows at processing.

Plant A had 28 positive cervical LN/472 tested (5.9%), whereas Plant B had 5 positive cervical LN/548 tested (0.9%) (Figure 1). The Plant A results are similar to that reported for inguinal LN prevalence rates of 37%, 8.9%, and 4.8% in sows and 6.4% and 13% in market hogs [9,10,11], whereas *Salmonella* prevalence for popliteal LN was 12.55% in market hogs [12]. Similar to the present study, those studies were in the U.S., whereas a study from abattoirs in Mexico reported 10.2% and 20% *Salmonella* prevalence in subiliac LN [13]. The decreased *Salmonella* isolation rates (0.73%) of Plant B in the present study mirror those of 0% reported for superficial cervical LN [17] and 0.06% for subiliac LN [18] of market hogs in U.S. plants. Although not peripheral LN, similar *Salmonella* prevalence results (6.1%) were reported for mesenteric LN from breeder sows in Spain [19]. 

For the 9-month testing period for mandibular LN, Plant A had 24 positive/150 tested (16%) and Plant B had 22 positive/217 tested (10.14%) (Figure 1). During the collection periods, Plant A had significantly increased *Salmonella*-positive cervical and mandibular LN samples when compared to Plant B (*p* < 0.05). Additionally, there were seasonal differences in *Salmonella*-positive samples with August having the highest number of *Salmonella* isolates when compared to the other months; however, this difference was not statistically significant when analyzing both sample types together (*p* = 0.22 (data not shown)) or individually for cervical and mandibular lymph nodes (*p* = 0.18 and *p* = 0.77, respectively (data not shown)). For whatever reasons, the second day of samples in Plant A and Plant B had numerically increased *Salmonella* isolates from cervical lymph nodes when compared to the results of the first day; however, this difference was not significant (*p* = 0.08 (data not shown)). In both plants, the percentage of *Salmonella*-positive samples were significantly increased in mandibular LN when compared to cervical LN (*p* < 0.05). This is not a great surprise in that lymphatic tissues that are close to the oral cavity (such as tonsils and parotid LN) tend to have greater *Salmonella* numbers when compared to peripheral LN sources [13,20,21]. Similarly, head trim and cheek meat tissues, anatomically close to the oral cavity, can have a high percentage (65%) of *Salmonella*-positive samples [22,23].

*Salmonella* isolates (64 tested) included 15 different serotypes: Adelaide (1), Agona (8), Alachua (1), Anatum (2), Anatum_var_15 (1), Berta (1), Brandenburg (2), Derby (25), Enteritidis (1), Heidelberg (1), Infantis (15), Johannesburg (1), Minnesota (1), Orion_var_15+34 (1), Typhimurium (1), and Uganda (1).

The majority (52.5%) of *Salmonella* isolates tested were pan-susceptible (no or intermediate resistance) to 14 antimicrobials; 37.8% were resistant to one class of antimicrobials, and six isolates (9.7%) were MDR. Two of the six MDR were serotype Brandenburg, one each from serotypes Infantis and Orion_var_15+34, and two isolates from Derby (Table 1). There were four MDR isolates from cervical LN and two from mandibular LN. These results are similar to *Salmonella* prevalence of 6.1% in mediastinal LN from sows at slaughter in Spain [19]. In that study, serotypes of isolates were: Typhimurium (43.7%), Derby (18.7%), Enteritidis (12.5%), and Montevideo (12.5%) with 9/16 of isolates showing multi-resistance to three or more antimicrobial classes (SSuT, ACSSut, and ASSut-Nx-Cfx). Although not LN tissue, the low levels of MDR in the present study contrast with a similarly designed study in which 63% of *Salmonella* isolates of cheek meat (anatomically close to mandibular LN) of market hogs were MDR [23].

## 4. Conclusions

On the basis of the results of this survey, it is evident that cervical and mandibular LN can be colonized by *Salmonella* and inclusion of these LN into pork products may increase the risk those products could be contaminated with *Salmonella*. Intervention methods to prevent foodborne disease could include elimination of these LN from pork products or inclusion of LN only into products that are destined for cooking. Additional research is needed to further understand the distribution and prevalence of *Salmonella* in the lymph nodes of commercial sows at slaughter. There is a high priority for the meat industry to utilize a multi-faceted approach to develop effective intervention methods to reduce *Salmonella* in the food chain.

## Figures and Tables

**Table 1:** Serotypes and antimicrobial resistance phenotypes of multidrug-resistant *Salmonella* isolates cultured from lymph nodes (LN) of sows at processing.

Isolate	LN ^a^	Plant	Month	Day	Serotype	Resistance ^b^
F8-1	C	A	Apr	3	Derby	STR, FIS, TET
I106-1	MD	A	Jul	10	Orion_var_15+34	AUG2, AMP, FOX, XNL, STR
J19-1	C	A	Aug	7	Brandenburg	FIS, TET, STX
J18EB1.1	C	A	Aug	7	Brandenburg	FIS, TET, STX
J104-2	MD	A	Aug	7	Derby	STR, FIS, TET
J51-2	C	A	Aug	8	Infantis	AUG2, AMP, FOX, XNL

^a^ C = cervical; MD = mandibular. ^b^ Antimicrobials.
